# Color, Scent and Size: Exploring Women's Preferences Around Design Characteristics of Drug-Releasing Vaginal Rings

**DOI:** 10.1007/s10461-022-03596-7

**Published:** 2022-03-16

**Authors:** Xinyu Zhao, Cecilia Milford, Jenni Smit, Bongiwe Zulu, Peter Boyd, R. Karl Malcolm, Mags Beksinska

**Affiliations:** 1 School of Pharmacy, Queen’s University Belfast, Belfast, BT9 7BL UK; 2 MRU (MatCH Research Unit), Department of Obstetrics and Gynaecology, Faculty of Health Sciences, University of the Witwatersrand, Durban, South Africa

**Keywords:** User-centred design, Pharmaceutical drug product design, HIV prevention, Contraception

## Abstract

Steroid-releasing vaginal rings are available for contraception and estrogen replacement therapy, and a new antiretroviral-releasing ring was recently approved for HIV prevention. Marketed rings are white or transparent in appearance, non-scented, and supplied as one-size-fits-all devices with diameters ranging from 54 to 56 mm. In this study, drug-free silicone elastomer rings were manufactured in different sizes, colors and scents, and the opinions/preferences of 16 women (eThekwini District, South Africa; 20–34 years) assessed through focus group discussions and thematic analysis. Opinions varied on ring color and scent, with some women preferring specific colors or scent intensities, while for others these attributes were unimportant. Concerns about color and scent were linked to perceptions around vaginal health and safety related to chemical composition. There was greater agreement on preferred ring size; flexibility and width were considered important factors for insertion and comfort. Greater choice with ring products could facilitate acceptability and overall uptake.

## Introduction

The concept of a polymeric vaginal ring device for use in sustaining drug administration to the human vagina was first described in the late 1960s [1]. Since then, seven ring products—mostly for hormonal contraception and estrogen replacement therapy—have reached market (Estring®, Progering® Fertiring®, Femring®, Annovera™, NuvaRing® and Ornibel®), and many other devices, including various generics and a dapivirine-releasing ring for HIV prevention, have been approved, are progressing through preclinical/clnical testing, or are  undergoing regulatory review for market approval [2–4]. In July, 2020, the European Medicines Agency (EMA) announced a positive regulatory opinion on the Dapivirine Vaginal Ring as an HIV prevention option for cisgender women aged 18 and older [5], and in March 2022 the ring received regulatory approval for use in South Africa.

**Fig. 1:**
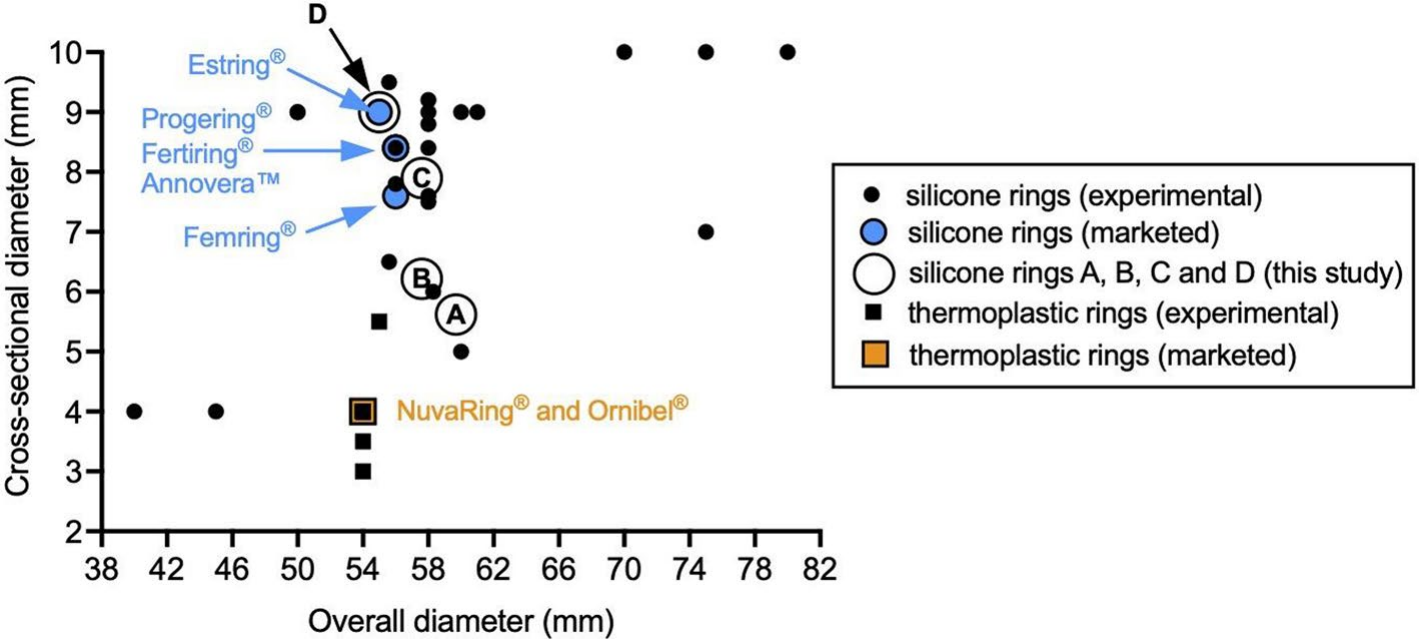
Dimensions (overall diameter vs. cross-sectional diameter) of vaginal rings, both marketed products (blue circles and orange squares) and those previously tested in the clinic and reported in the scientific and patent literature (black circles and squares). Note that rings are further sub-classified according to polymer type—silicone elastomer (circles) and thermoplastic (squares). Large white circles represent the drug-free rings used in this study

Despite considerable variation in baseline vaginal dimensions [6], all marketed drug-releasing vaginal rings are designed, manufactured and supplied as one-size-fits-all devices. Drug-releasing vaginal ring devices with external diameters ranging between 38 and 80 mm have previously been tested in women, although marketed devices are mostly limited to one of three external diameters—54, 55 or 56 mm—irrespective of the polymer type used to construct the ring (Fig. 1) [2]. There is greater variation in ring cross-sectional diameters (4–9 mm), with silicone elastomer rings falling within the upper end of the range (7.6–9.0 mm) and thermoplastic rings (such as NuvaRing® and Ornibel®) fixed at ~ 4 mm (Fig. 1).

**Fig. 2:**
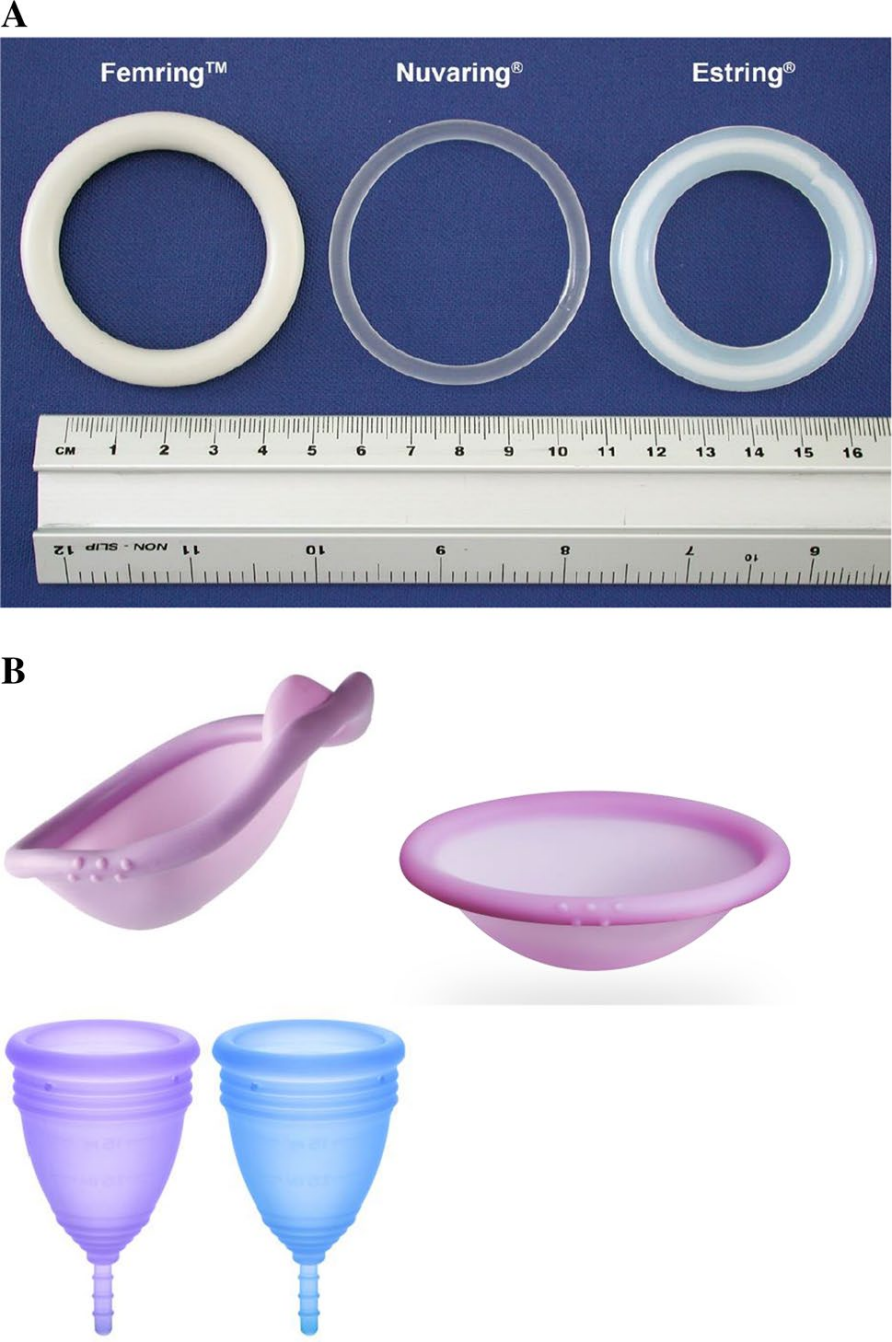
**A**—Examples of marketed vaginal rings, showing conventional white or transparent/colorless appearance. **B**—Examples of colored products administered vaginally—Caya® and Singa® diaphragms, and menstrual cups

In general, it is well established that the color of any product—including drug products and medical devices—can induce emotional and psychological responses, with preferences varying across age, time, culture and product type [7–10]. Within the pharmaceutical industry, color may be useful in avoiding medication/dosing errors for products that might otherwise look similar. Historically, many medical devices and pharmaceutical products have been colored white, widely considered to signify cleanliness and purity. However, from the perspective of chromology (the study of color psychology), white products are also considered weak and may also reinforce the negative stereotype and impersonal perception associated with medical and drug products [7]. To date, this traditional color strategy has also been applied to all marketed and experimental drug-releasing rings, which are either opaque white or transparent and colorless in appearance (Fig. 2). This contrasts strongly with oral dosage forms which often contain highly colored film coatings to improve product appearance and identification, and provide opportunities for enhanced branding and trademarking [7, 11, 12]. Ring-shaped vaginal pessaries for treating pelvic organ prolapse—in addition to being available in a very wide range of sizes (overall diameter 38 to 127 mm [2]) —are also supplied in a selection of colors, including white, pink, orange and blue [13]. Discoloration of white-colored rings—both drug-releasing vaginal rings and ring pessaries—during use has also been reported [14–16]. This discoloration is attributed to adherence of components of cervicovaginal fluid and menstrual blood to the ring surface, although it is considered not to affect clinical outcomes.

To date, there has been only limited clinical evaluation of ring size [17–21], despite the likelihood that providing options to women around these product attributes could significantly enhance user acceptability and, in turn, product adherence and efficacy [22, 23]. A small number of studies have reported women’s perceived preferences for ring size, but no study has presented women with different color and scent options for assessment. In one randomized controlled trial and in two observational studies, 61.4–85.7% of users reported that a vaginal ring diameter of 56 to 58 mm and a cross sectional diameter of 7.7 to 8.4 mm were acceptable [23–25]. Some women have stated they would prefer the ring to be thinner, and a few would prefer the ring to be more flexible [22]. Size preference may be related to women and their partners feeling the ring during sex: studies report that 70–90% of users and 48–97% of partners felt the ring during sex [26]. Acceptability of most ring attributes increases in with duration of use [23, 26].

Studies show that consumers—and patients using pharmaceutical products—form an emotional link with color, either associated with the product itself or its packaging [27–32]. Despite this, most drug dosage forms are white. The TRIO study aimed to determine ratings and preferences of three (placebo) delivery forms (oral tablet, intravaginal ring, and intramuscular injection), among young women in two sub-Saharan African communities, and found that two-thirds of women reported the white placebo vaginal ring looked acceptable [23]. In another study, participants had different perspectives on perceived ring color [22], with some liking the white ring color, because it was seen as simple, plain, or looked “medical grade.” Others preferred colored rings (e.g., purple, pink, blue, multicolor, glow-in the-dark, “fun colors”) to make the ring look more fun, “less sterile,” more natural, or more “feminine”. However, some women were concerned that use of a dye to give the ring color may affect health. Color was less important for some participants as the ring was not seen once inserted [19].

Generic products that incorporate innovative branding strategies, such as color, often have higher market share than plain white tablets (e.g. Quetiapine 25 mg, Levetiracetam 750 mg) [33]. Many approved and marketed vaginal products are now available in a color option including menstrual cups, female condoms and a vaginal diaphragm [34]. In a study that assessed choice of female condom color when offered a choice of plain/unscented, and two colored/scented options, almost all women chose the colored/scented condoms [35].

In this study we explore women’s preferences around three vaginal product attributes considered important for increasing user acceptability—size/dimensions, color, and scent. In addition to exploring these preferences for existing vaginal products available in these different attributes, we designed and manufactured drug-free silicone elastomer vaginal rings having different sizes, colors and scents. We conducted three focus group discussions (FGDs) with women in eThekwini District, KwaZulu-Natal, South Africa, to explore preferences and attitudes to different vaginal product attributes (size, color and scent), with a specific focus on vaginal rings.

## Materials and Methods

### Materials

Medical grade addition-cure silicone elastomer MED-4870 and various color masterbatches for liquid silicone elastomers (consisting of pigments dispersed in a vinyldimethyl-terminated polydimethylsiloxane polymer; MED-4900-3—red; MED-4900-4—orange; MED-4900-5—yellow; MED2-4900-6—light green; MED1-4900-7—blue) were obtained from NuSil Silicone Technology Inc. (Carpinteria, CA, USA). Silbione® Biomedical LSR D1XX-TB silicone elastomer (Batch No. 07117-15) was obtained by Elkem Silicones (South Carolina, USA). Titanium dioxide was obtained from Sigma-Aldrich (Gillingham, UK). Essential oils, for ring scent (Lavender True 102; Lemon Organic 103; Grapefruit Organic 115; Camphor White 133; Spearmint Organic 165), were obtained from Naissance (Neath, UK).

### Manufacture of Silicone Elastomer Vaginal Rings Having Different Colors

**Fig. 3:**
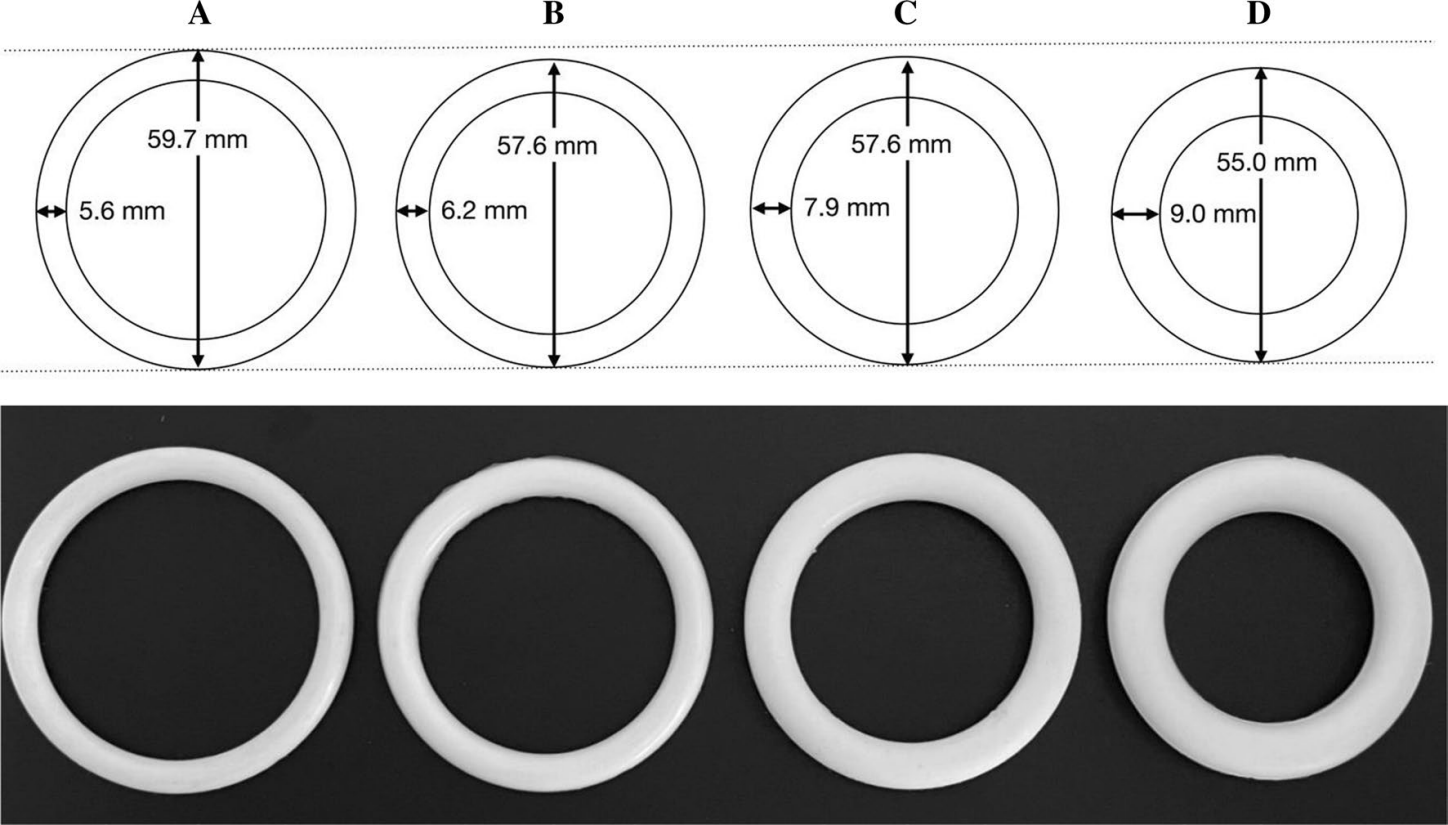
Dimensions (cross-sectional and external diameters; drawn to scale) and photographs of the four different ring designs used in this study. See Fig. 1 for comparison of ring dimensions with those of marketed and experimental rings

**Fig. 4:**
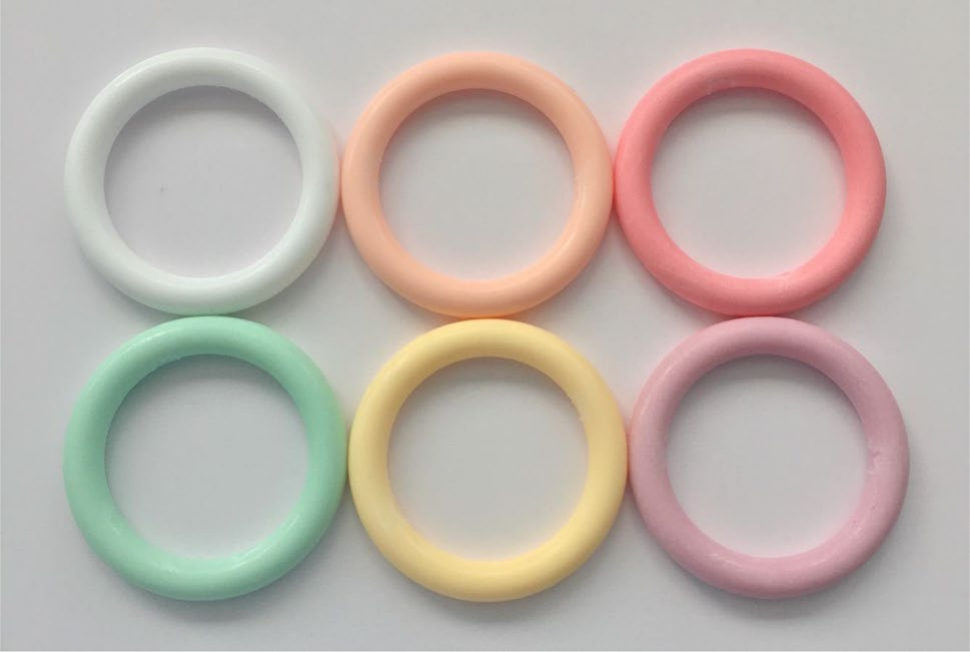
Ring colors from top row left to right—white, pastel orange, light pink; bottom row—pastel green, mellow yellow and mauve. See also Table 1

Non-medicated vaginal rings having six different colors—white, mellow yellow, light pink, pastel orange, pastel green and mauve (Fig. 3)—were manufactured using a Babyplast™ 6/10P injection molding machine (Chronoplast, Spain) fitted with custom 57.6 × 7.9 mm ring molds. Briefly, titanium dioxide (a non-active, pharmaceutical grade excipient to simulate the white color typical of pharmaceutical drugs) and various medical grade color masterbatches were mixed with Parts A and B of MED-4870 silicone elastomer at specified concentrations (Table 1) using a DAC-150 FVZ-K Speedmixer™ (Hauschild, Germany; 3000 rpm for 30 s). Part A and B mixes were sequentially added to a large plastic polypropylene Speedmixer container until ~ 400 g in total had been transferred. The contents were mixed with a spatula (10 s) and then using a DAC-600 Speedmixer™ (Hauschild, Germany; 1500 rpm for 30 s). The contents were then transferred into a modified E1000 cartridge (Fiscbach, Germany). The cartridge was inserted into a cartridge holder, the assembly fitted in the Babyplast™ machine, and rings manufactured at 160 °C for 90 s using predefined injection molding parameters optimised for this elastomer: shot size (7.70 g); first and second injection pressures 50 and 15 bar, respectively; clamping pressure 100 bar. Vaginal rings were then demolded and heat-sealed in aluminium foil pouches (Fig. 4).

**Table 1:** Silicone elastomer vaginal rings (dimensions 57.6 mm overall diameter and 7.9 mm cross-sectional diameter) having different colors were obtained by incorporating various concentrations of titanium dioxide and color masterbatches into MED-4870 silicone elastomer

Ring color	Titanium dioxide concentration (% w/w)	Concentration of color masterbatches (% w/w)
White	0.4	–
Pastel orange	0.4	0.02 MED-4900–4
Light pink	0.4	0.05 MED-4900–3
Pastel green	0.4	0.10 MED2-4900–6
Mellow yellow	0.4	0.01 MED-4900–4 + 0.02 MED-4900–5
Mauve	0.3	0.02 MED-4900–3 + 0.04 MED1-4900–7

### Manufacture of Silicone Elastomer Vaginal Rings Incorporating Scents

Careful selection of scents for incorporation into rings is necessary, given (i) the propensity of certain functional groups within molecules to react with silicone elastomer materials [36–38], (ii) the potential for evaporation of volatile organic scent molecules during high-temperature manufacture of the rings; and (iii) the risk of mucosal irritation with certain scent substances [39]. Scents are already added to certain marketed condoms and vaginal wash products.

For the purposes of this study, we selected Silbione® Biomedical LSR D1XX-TB silicone elastomer and five essential oils for incorporation at very low concentrations into rings during manufacture (Table 2); the final oil concentrations were selected based on manufacture and testing of prototype rings having a broad range of concentrations. Rings were manufactured by injection molding (curing conditions: 112 °C for 100 s) using a method similar to that described in Section "Manufacture of silicone elastomer vaginal rings having different colors".

**Table 2:** Silicone elastomer vaginal rings (dimensions 57.6 mm overall diameter and 7.9 mm cross-sectional diameter) having different scents were obtained by incorporating various essential oils into Silbione® Biomedical LSR D1XX-TB

Ring scent	Essential oil concentration (% w/w)
Lemon	5.0
Grapefruit	3.0
Spearmint	2.0
Camphor	1.0
Lavender	0.50

### Manufacture of Silicone Elastomer Vaginal Rings Having Different Sizes

Vaginal rings having four different dimensions (A—57.7 × 5.6 mm; B—57.6 × 6.2 mm; C—57.6 × 7.9 mm; D—55 0.0 × 9.0 mm; Fig. 3) were manufactured from medical grade addition cure silicone elastomer MED-4780, using custom ring molds fitted to an electrically-heated, laboratory-scale injection molding machine. Part A and Part B MED-4780 silicone elastomer premixes containing titanium dioxide were prepared using the method described in Section "Manufacture of silicone elastomer vaginal rings having different colors". The mixtures of A and B were then transferred to a SEMCO 2.5 oz HD cartridge, injected using a DCG180 Makita caulking gun into custom molds having different dimensions, and cured at 160 °C for 90 s.

### Focus Group Discussions

We conducted qualitative research via focus group discussions (FGDs) to explore women’s preferences and attitudes to different vaginal product attributes (size, color and scent), with a focus on the vaginal ring. The study was conducted in the eThekwini District, KwaZulu-Natal, South Africa, during March 2021.

Three FGDs were conducted with women aged 18–35 years. Women who had previously participated in research studies/clinical trials at the study site, and who had consented to future contact, were purposively selected and invited to participate in a single FGD. Selection included a range of women with and without vaginal product experience, and this information was used for setting up the three groups as follows: (1) women who had experience with vaginal products, (2) women who were naïve to vaginal products, and (3) women with varied vaginal product experience (both experienced and naïve product users).

Basic demographic information was collected from participants prior to the discussion. The specially manufactured vaginal rings of varying colors, dimensions and scents, described above, were made available in the groups for women to visualize, handle and smell. Other marketed vaginal products (diaphragms, cervical cups, colored/scented female condoms/menstrual cups and tampons) were also made available to facilitate size, color and scent comparisons. Rings and other products were not inserted or used.

All three FGDs were conducted in isiZulu, the preferred local language. The discussions were audio recorded with the consent of the participants, transcribed, and translated from isiZulu into English. Qualitative data analysis software (NVivo v12, QSR International) was used to organize, code, and analyse the data. Based on inductive and deductive coding, the FGD data were thematically analysed. Demographic data from participants was captured on REDCap [40] and descriptive analyses were conducted.

### Ethical Considerations

The study was approved by the Human Research Ethics Committee (HREC) of the University of the Witwatersrand (M200968). All women provided written informed consent prior to participation, with separate consent for the audio recording of the discussion. Consenting was conducted in isiZulu or English according to participant preference.

## Results

### Participant Characteristics

A total of 16 women participated in the three FGDs. At project inception it was anticipated that up to 10 women would participate in each FGD. However, due to COVID-19 social distancing restrictions, each group was limited to five or six women.

Participant socio-demographic information and previous vaginal product use experience is shown in Table 3. Women had a mean age of 26.8 years. The most commonly reported source of household income was through the South African (SA) government grant (n = 15), followed by some income from various levels of employment (n = 11). Women had a range of experience with vaginal products, mostly in research studies in which they had previously participated. All participants with vaginal product experience had used at least one of these products in a clinical trial. No woman had used a menstrual cup.

**Table 3:** Demographic characteristics of focus group participants

Demographics	(N = 16)
Age (mean, range)	26.9 (20–34)
Ever pregnant (%, N)	75.0 (12)
Number of pregnancies (mean, range)	1.8 (0–5)
Source of household income (%, N^a^)	
Employment (full/part time/casual)	68.8 (11)
Government grant	93.8 (15)
Financial support from outside household	18.8 (3)
Education (grade) (%, N)	
Secondary incomplete	12.5 (2)
Completed secondary	68.8 (11)
Tertiary	18.8 (3)
Current relationship (%, N)	
Regular partner, not living together	75.0 (12)
Regular partner, living together	18.8 (3)
No current relationship	6.3 (1)
Vaginal product experience* (%, N)	
Vaginal ring	50.0 (8)
Female condom	43.8 (7)
Vaginal applicator	43.8 (7)
Tampon	37.5 (6)
Vaginal tablet	37.5 (6)
Vaginal film	31.3 (5)
Diaphragm	6.3 (1)
Menstrual cup	0.0 (0)

^a^Multiple choice options

All participants in FGD Group 1 had personally used one or more vaginal products, and had experience using a placebo vaginal ring in the Quatro study, which determined ratings and preference for four (placebo) vaginal delivery forms (tablet, intravaginal ring, film and gel) [41]. Although all participants in FGD Group 2 were recruited on the basis of having reported no previous vaginal product experience, two of the six participants in the group had previously tried to use a tampon but had not continued to use them, while one woman had tried unsuccessfully to use a female condom. These three women only mentioned their unsuccessful attempts to use these vaginal products when the FGD was in progress. Similarly, in the mixed experience group (Group 3), two of the women who had been recruited as inexperienced vaginal product users had also previously tried to use a female condom but had not used it during sex. Two women in the mixed experience group had used the placebo vaginal ring in the Quatro study [41].

### Thematic Results

The thematic findings results are presented according to preferences and attitudes for vaginal product attributes, specifically: color, scent and size. In addition, vaginal ring use and virginity testing practices are discussed. Where quotations are provided, P refers to a participant and F to the facilitator.

#### Color

##### Color Preference for Vaginal Products

There was agreement across FGDs that (i) product color choice was an individual decision, (ii) some people prefer colored products while others prefer white/transparent products, and (iii) in some cases offering a choice of various colors is preferable.

“I think women would prefer colors, but also have products without color because we are not the same as women, there are those who are out there, who may tell you that they prefer the colored ones, like [other participant] has mentioned. I think if that happens, we should have all of them mixed, so they can choose.” (Group 1, Participant 1, product experienced group)

“I think colors are very attractive. If you are told that this product is for women, I think the first thing that attracts you is color before you even learn how it works. Even if you get inside the shop, you might just feel it, even if you do not plan to buy it. You just look at this thing with a beautiful color and you start asking about it if you do not know about it, and they explain it to you and then you buy it, but the color is very attractive.” (Group 2, Participant 2, product inexperienced group)

Participants in Group 2 noted that the efficacy, safety and comfort of the product was more important to them than the color.

“Ehh, honestly, I do not have problem with color, even if it is white, transparent or black. […] The effects and how they are used only that are important to me. I do not have a problem with the color.” (Group 2, Participant 6, product inexperienced group)

“Just to add, color is not important more than safety and comfortability. […] as long as there is safety and I am comfortable, I think that is enough for me.” (Group 2, Participant 2, product inexperienced group)

Some participants had concerns that color could cause vaginal irritation, and for this reason they preferred products without color.

“I do not have a problem with color, as long as they do not have any chemicals.” (Group 3, Participant 2, mixed experience group)

“I also prefer colorless. […] My reason is that in most cases when the product is made… […] There are things that are added, that may cause another person to have allergies when using them and things like those. So, I prefer them to, even though they add some things, to make sure everything is hygienic. But I am sure there are things that are added, but they should not cause infection.” (Group 3, Participant 4, mixed experience group)

Participants also described varied preferences for their male partners when it came to color of products. Some felt their partners would prefer products without color, although some said their partners preferred colored products, for example condoms.

“I think men would prefer the ones without color. […] Men like simple things than colors. Some of them do not like colors, they might think it has some flavours.” (Group 1, Participant 4, product experienced group)

“I have noticed that a lot of times they [men] like the colored ones [referring to condoms].” (Group 1, Participant 2, product experienced group)

A few participants felt that their male partners would be suspicious of products with color and described concerns of witchcraft.

“Another thing is that since it is colored, maybe it might happen that it might be visible or maybe you can tell your partner that you use things like these. He might ask if these colors do not have, there are people who believe that people use bewitching medicines and they are always vigilant of those bad things, he might ask what you have put here for him, what are these colors for. So, it is alright in a white color because even if he sees it, he will not be surprised that much.” (Group 1, Participant 5, product experienced group)

P: “You see, honestly, men, oh my, they are not educated. He would say, “What do you have on now? Take this thing off”. Honestly, my partner would not like any of them, I am just talking about my partner, he would ask, ‘What have you inserted?’.”

F: “Okay, so, there is not one he would like from these colors? Even if it is a color that is not included here?”

P: “I am telling you that anything he would see inserted in my vagina. The other fear they have is that they just wonder what you have inserted.” (Group 2, Participant 1, product inexperienced group)

##### Ranking of Vaginal Ring Colors

Participants were shown six vaginal rings of different colors/shades (including white, mellow yellow, light pink, pastel orange, pastel green and mauve) and asked which color they preferred (Table 1, Fig. 4). Group 1 participants largely preferred the white vaginal ring, one suggesting that the white color would enable her to detect any discharge/disease. Although some participants in the group noted that different people would have different preferences, and suggested individuals should be able to choose their preferred option.

“Because white might help me to see if there is some dirt in my vagina, be able to spot discharge, but the coloured ones, no. I like it like this.” (Group 1, Participant 1, product experienced group)

“I think all the colors that are here are not a problem. I like all of them, and then have a person choose which one they like. There is no need to add more, the ones that are available are fine. A person can decide which color they like.” (Group 1, Participant 2, product experienced group)

Participants from Groups 2 and 3 chose a range of colors related to individual preference.

F: “Okay, number 1 chose mauve, 2 and 3 chose peach [referring to pastel orange], 4 chose white and 5 and 6 chose…”

Note-taker: “Lime (pastel green).” (Group 2, product inexperienced group)

Furthermore, some participants suggested a few additional colors be added to the options including yellow, pink, black and red. Although yellow (mellow yellow) and pink (light pink) rings were included in the study, yellow and pink may have been mentioned as additional colors as the intensity of the study colors was not very strong.

P: “I think black as well, people like color black.”

F: “You think people like black and you think they can use black?”

P: “Uhm hum. I think they can love it.”

F: “Okay. Can you tell me why you think people might like black?”

P: “Uhm, black is calm, so, ya.” (Group 1, Participant 3, product experienced group)

##### Preferred Intensity of Colors

Participants in all groups felt that the pale colors of the sample vaginal rings were acceptable for various reasons, including that their partner may not be able to see it, it was perceived as safer (fewer chemicals added), and for personal preference.

“So, we do not like, women like me, do not like the intense colors, sweet [bright] pink would feel like it is for young people because young people like sweet colors, you see that things that are going to be attracted to. Unlike us, matured women, you know that you prefer a calm color.” (Group 2, Participant 1, product inexperienced group)

P: “Ay, the colors are alright like this.”

F: “They are good as light as they are. What makes you like the light colors number 2.”

P: “Because once it is dim, it might look as though something is added.”

F: “Something like what?”

P: “Something like chemicals.” (Group 3, Participant 2, mixed experience group)

“Okay, if the rings are light, it will not be easy for him to notice that I have something on […] Whereas, if it is intense, it is out of the original color of your vagina, it will be visible and that can make him uncomfortable, even if he is aware that you have it on neh, but when he sees it and see where it is sitting, it would be uncomfortable for him to penetrate without him feeling like he is going to hurt you, you see.” (Group 3, Participant  unknown, mixed experience group)

However, some participants in Group 2 felt that brighter, more intense colors would be preferable for some people.

“I like sweet [bright] pink, I like the intense colors, there is no place written that sweet colors are for kids. [*Some participants are laughing*] Because I have a sweet-pink jacket, I have a sweet-pink clothes, so now I cannot wear them? [*Laughing*].” (Group 2, Participant 6, product inexperienced group)

The same participant felt that light-colored vaginal rings could become darker over time, and the darker color could be used as an indicator to detect ring safety issues:

“The way I see it is that the reason they are light, you insert this ring and remove it after some time, right? […] Maybe when it is not right, it becomes darker, when it is expiring. That is the way you are able to see, that it becomes darker.” (Group 2, Participant 6, product inexperienced group)

#### Scent

##### Preference for Scented Versus Non-scented Vaginal Products

Participants had a range of preferences regarding scented/unscented vaginal products, with some not liking to use scented products.

“Ey, I do not like them [referring to the SA government colored/ scented male condoms], others have scents and I do not like scents, and I end up not enjoying sex because of the smell.” (Group 1, Participant 4, product experienced group)

Others described that they preferred products with scents. Many of them felt that the scented products masked the smell of sex or the smell of the vagina.

“I like the colored ones [referring to the SA government colored/scented male condoms] because they cover the sex smell, you just smell strawberry or banana, but the non-colored ones, you can smell sex as it is.” (Group 1, Participant 1, product experienced group)

“Do you know when you are busy having sex, SEX, [*Participants laughing*], there is that smell, the vagina has its own smell, sex on its own has its smell, so, I think, as I said that these, as I explained that since lemon is this strong and nice. Even a person coming in the room after you had sex, they will not feel that you were having sex, they will not feel that smell, that sense of saying, “Eish there was sex happening here”, there are people who have smelly vaginas, you see. But if lemon flavoured ring is inserted, when the partner penetrates you, he will just smell lemon. He knows that my vagina smells lemon, you see. [*Participants laughing*]. It smells like spearmint, you see. It is not that spearmint flavour will be the same as Chappies (bubblegum), a strong Chappies, because I think they would not create something for us to insert that is going to be strong as Chappies inside our vaginas. It is just that flavour, that smell of that certain flavour.” (Group 2, Participant 1, product inexperienced group)

Some participants suggested that people should have a choice of whether a product has a scent or not.

“I would prefer to have both, flavoured and non-flavoured [meaning scented and non-scented], so that a person can take their choice.” (Group 3, Participant 2, mixed experience group)

Participants in all groups suggested that their concerns around product scent were related to whether the scent could cause vaginal irritation:

“I also do not see the need for the scent because some of women are sensitive in their vaginal areas, these scents are very strong. I might insert the lemon scented one and it does not like me, and maybe give me rash, so, I would just add color, but not the scent.” (Group 1, Participant 1, product experienced group)

When asked whether their male partners would prefer products with a scent or not, responses also varied. Some also suggested that their partners preferred using scented products to cover the smell of sex.

“I can say my partner likes us to use the ones with color [referring to the SA government colored/scented male condoms] and I do not like them, but he says it is because of the room, it does not smell, there is that smell that says you are having sex. […] But if we use the colored one, that smell is not there, it just smells nice, but for me, I do not prefer it, it does not smell good for me.” (Group 3, Participant 1, mixed experience group)

One participant was also concerned that the scent may also irritate/have health implications for her male partner.

“I also wanted to say something like that and what I also wanted to add is that maybe the scent might not give me any problems and does not give me rash, like they have mentioned, but my partner might have problems. That is what I think.” (Group 1, Participant 5, product experienced group)

##### Ranking of Sample Scents

Participants were provided with sample vaginal rings with different scents—lavender, grapefruit, lemon, spearmint, camphor (Table 1)—and were asked which ones they preferred. Participants in the different groups ranked products differently. Group 1 preferred lavender and grapefruit, as they felt the scents weren’t as strong. Overall, in Groups 2 and 3, lemon and spearmint were preferred, with some participants also suggesting a preference for lavender. Preferences appeared to be related to personal associations with the scents.

P1: “Ehh, for lavender, let us start with lavender, it is like you cleaning the house. [*Participant laughing*]. We would be busy having sex and the scent of cleaning a house is around us, no. [*Participant laughing*]. And then…grapefruit, I also did not like it because it has that orangey flavour to it. Ehm, what else? Camphor, awe! He is going to ask me if I use camphor because it smells exactly like camphor, a body lotion. I do not use camphor and now he finds it in my vagina, no. […]”

P4: “I like lemon and lavender, the lavender for me smells like perfume. I did not feel like it is a cleaning product. […] I do not like camphor.” (Group 2, product inexperienced group)

“I also like lemon. It smelled nice; it has that refreshment to it. Ay, when I smelled spearmint, Ha! It is too strong. I know Chappies [bubblegum] smells like this, not if it is going to be my vagina smelling like this, it is too much, it is too much for me. […] Ya, spearmint is too much for me, it is very strong, but lemon also strong but it is nice, it has that refreshment to it.” (Group 2, Participant 6, product inexperienced group)

Some participants had concerns that some of the stronger scents would cause vaginal irritation, so didn’t rank them as highly:

“[S]pearmint and camphor, have a stinging feeling to them even with the smell, you know when you eat something that is spearmint flavoured, it stings. So, I would prefer lemon.” (Group 3, Participant 3, mixed experience group)

##### Preferred Intensity for Scents

Participants had varied opinions about the intensity of the sample scents. Some (in Group 1) felt that the sample scents were too strong:

“[T]hey are very, very, very strong. […] Too strong, yes.” (Group 1, Participant 3, product experienced group)

Others felt some of the sample scents were too strong, and other scents were very weak:

P: “Ehh, the lemon one is quite strong, when I opened it, the scent overcame me, so I am imagining opening it in the house and you are having sex, now the whole house smells like lemon.”

F: “So, you do not like lemon?”

P: “No, I like it, I am just saying it is too strong to the point that it is great because the house will smell more like it.”

F: “So, you like how strong it is?”

P: “Yes, I like its strong scent as well as the spearmint. […] Ya, it also smells good, it has enough scent. And then camphor and lavender I think they have a weak scent, yes and [grapefruit] I only smell a little bit of it, I do not smell them they do not attract me but the other lemon and spearmint, I like best.” (Group 2, Participant 2, product inexperienced group)

##### Duration of Scent

Participants from all groups felt that it would be best if the product scent did not fade with use or over time. This was largely related to concerns about vaginal hygiene and masking of the vaginal smell.

“I think…it would be better to have the scent even on removal because if the scent is gone when removing it, you would ask yourself where it went and you would think it stays inside you, you see. It is better to have it throughout, until you remove it.” (Group 1, Participant 2, product experienced group)

“I think if we had scent throughout… […] And then it depends on how hygienic a woman is. Let us say she is dirty, and she inserts this product, and the scent is there throughout, the scent might cover the unhygienic smell from her, but when it is removed, that unhygienic smell from a woman comes back and covers the scent from the ring. So, I feel that even if it have scent, it would depend on how clean the woman is, if she is clean, the scent can stay until the ring is removed, but if she is not hygienic, the scent may not last and when the ring is removed, it comes out dirty because of the lack of hygiene.” (Group 3, Participant 1, mixed experience group)

One participant in Group 3 felt that it would be best for the scent to only be there on insertion, firstly so that the scent did not cause vaginal irritation, but secondly so that the partner did not smell it, in the case of covert product usage.

“I would prefer it to have scent when it is inserted only because I do not know if it is going to harm me if the scent stays inside me, you see. […] Also, some people, they might insert the ring, and not tell their partner that they have it on and they remove it. Say now the boyfriend comes and sucks you and tastes this scent, coming from inside you. [….] So, it is better to have it scented only when inserted and have the scent disappear inside you and not have scent when removed.” (Group 3, Participant 2, mixed experience group)

#### Vaginal Ring Size

##### Ranking and Preference of Vaginal Ring Sizes

Participants were shown four vaginal rings of different dimensions (Fig. 3). Overall, participants from all groups stated that they preferred the size of vaginal ring A. They felt that it would be easier to insert, and more comfortable to wear.

“I also feel that A is perfect because can you see it, it is wide and small compared to D, D. It’s (referring to D) scary, even looking at it is scary, it might go and sit in the wrong places and maybe end up not coming out or do harm. […] You know, so really, like A is perfect.” (Group 3, Participant 1, mixed experience group)

Some participants expressed preference for other vaginal ring sizes:

“Okay, I chose B, the reason why I chose B is that…okay, the size is almost the same as A. […] But what I like about it is that it is not wide, you cannot just insert something big in my vagina. I can be able to pinch in and it will not open wide your vagina. Now A makes me feel like it is too wide. [*All Laughing*] It is too wide, you see. Whereas, C and D are hard, I would not be comfortable. So, I think B is soft and it will not open my vagina that wide.” (Group 2, Participant 1, product inexperienced group)

However, one participant did note that it was difficult to choose without actually having tried to insert/use the different vaginal rings:

“The problem is that we do not know, because we have never used them and we do not have experience, but A is right.” (Group 3, Participant 1, mixed experience group)

Participants were also asked which vaginal ring size option they thought that their male partners would prefer. Preference was related to impact the vaginal ring would have on sex—whether it fit onto their partner’s penis or not (possibly related to a misconception that the penis should fit through the vaginal ring), or if ring use widened or tightened the vagina. Ring size preference varied, participants largely felt their partner would prefer ring size A or D.

“I think he might like D. […] D is not as wide, I am still standing on that, even if he inserts his penis, it would move just a bit, but with this, he might say, ‘No baby, this is a canoe’ (meaning that the inside of the vagina is even bigger now that the ring has been inserted).” [*Participants laughing*] (Group 2, Participant 1, product inexperienced group)

P: “I like A and I see that I can be comfortable because even if it sits like this inside, you see, but when it comes to sex, no, it is big, I choose D.”

F: “Okay, let me ask one question. Let us say, he would not feel it. If once it is inserted and positioned correctly, and your partner does not feel it because I, personally do not know, you see, but if you cannot feel it, which one would you be comfortable with?”

P: “I would be comfortable with A, if he cannot feel it and if it sits in a correct place and my vagina is not affected, where the penis enters, then, I would choose A.”

F: “Which one do you think your partner would like?”

P: “If he will not feel it?”

F: “If he does not feel it, as much as he would know that it is there but does not feel it.”

P: “It is A.”

F: “He would choose A as well?”

P: “If it not going to affect my vagina, but if it makes my vagina stretch, then, it is D.” (Group 2, Participant 6, product inexperienced group)

“I just think that it depends on a man’s manhood size. Maybe for one might say D is right and the other one might say B is right.” (Group 3, Participant 1, mixed experience group)

##### Insertion and Removal, Comfort During Use

Participants talked about challenges/ease with vaginal ring insertion and removal when discussing vaginal ring size preference.

“[Y]ou see, this is too big (referring to C), I foresee problem when trying to insert this one. Seriously, it would be a problem. […] We are not used to this size and now if we have to insert this size, I would honestly never insert it.” (Group 1, Participant 2, product experienced group)

“Okay, I chose B, the reason why I chose B is that…okay, the size is almost the same as A. […] But what I like about it is that it is not wide, you cannot just insert something big in my vagina. I can be able to pinch in and it will not open wide your vagina. Now A makes me feel like it is too wide.” (Group 2, Participant 1, product inexperienced group)

Preference for the vaginal ring sizes was also related to perceived comfort during use:

“You can see that A is not the same size as the rest of the rings, others are a bit bigger than A. So, A was created so that when it is inserted it sits well and you feel comfortable.” (Group 1, Participant 2, product experienced group)

##### Vaginal Ring Dimensions and Properties: Width/Thickness, Diameter and Flexibility

Smaller rings were preferable because they were perceived to be easier to insert.

“[B]ut A, it most people’s preference because it is small and flexible, so I think they decided to choose something that the majority of people might like at that time.” (Group 3, Participant 3, mixed experience group)

Thicker rings were perceived as less comfortable, and more difficult to insert.

“[T]alking from experience, I have tried to insert the female condom, you know guys, size D, it is really difficult to insert this as thick as it is, but A is simple, you are able to push it. Imagine inserting the D and it gets stuck in the vagina before you can even push it to reach the top, because you have to push it, I think you have to push it until it reaches the top, like a tampon. How is this thick thing going to be inserted?” (Group 2, Participant 6, product inexperienced group)

“You see the D size one… […] When we got the ring, we were told how it is inserted, you need to pull and hold it like this, you see when I do this, it is hard for it to fold, which might happen that after inserting it, it goes and sits in a wrong way. […] I am talking about its flexibility. […] I wish it could be like this one. [...] It is A.” (Group 3, Participant 4, mixed experience group)

Some participants preferred covert ring use, and were worried their partners would feel the thicker rings during sex.

“A is alright all the way. It is thin and wide, but C and D are thick and tight. Like I have mentioned earlier on that we hid from our partners that we were using the ring [in the Quatro study], it was not easy for him to feel A, but C and D he could have felt because they are thick.” (Group 1, Participant 1, product experienced group)

Participants felt that the more flexible rings would be easier to insert and more comfortable to use.

“Ahh, [Ring A] it is soft. I think it will be flexible to turn it when you insert it, maybe it could be easy, and it is stays in your uterus, it will not be as hard and tense to the point that you cannot even do anything, yes, it looks comfortable.” (Group 2, Participant 6, product inexperienced group)

Although, one person felt that the vaginal ring could possibly be too flexible:

“I would say B (is preferable) because A is too thin and too flexible, I would prefer it to be B because it can bend and is a bit thick, you see, ya.” (Group 3, Participant 3, mixed experience group)

One participant thought perhaps the thicker rings had been designed so that sperm could not pass through:

“I am looking at its thickness, maybe it is made so that the sperm does not enter where the eggs are. Is that not why?” (Group 2, Participant 4, product inexperienced group)

#### Vaginal Ring Use and Virginity Testing

Some participants described that the practice of virginity testing was still being conducted in their areas. They noted that product insertions, such as vaginal ring or menstrual cup use, could tamper with the hymen and impact the virginity testing process. However, some did say that virginity testing should be a reflection of intercourse, and not product use.

I think these products touch there, because a virgin has to always be tight, right? I know that you lose your virginity once the penis enters your vagina. Does these products not affect ones virginity. Some of these things poke, you see. Does it not open you there, the inside of the vagina hole? (meaning does it affect your virginity) That is my concern that, I have a daughter who is about to go on her periods, imagine me telling her to use these cups (referring to menstrual cups). Will they not touch the hymen that determines her virginity and ruins it every time? (Group 2, Participant 1, product inexperienced group)

No, I do not think she would have a problem (with virginity testing) because she knows that a penis has never penetrated her. What she is using is something to prevent her from getting HIV […] So, she knows that she has never been, even though there is that logic that these things affect it. […] But deep inside she knows that she has never been penetrated. (Group 2, Participant 1, product inexperienced group)

## Discussion

Participants had a range of vaginal product experiences, primarily from previous participation in research studies/trials at the research site. For this study, each participant was given a set of vaginal rings of the different colors, sizes and scents, plus a range of other vaginal products in white/transparent and colored versions of the products. Women were able to handle and look at these products during the FGD discussions. They did not use or insert the products.

Similar to other research, opinions on vaginal ring color were varied with some women clearly preferring colored products while for others this was not an important attribute [22]. Some women raised safety concerns related to color in vaginal products possibly causing vaginal irritation. There was an assumption that the colors may contain chemicals and women felt that there should be reassurance that insertion of colored products into the vagina would be safe. Safety, comfort and efficacy was seen by some women as more important. In a study on vaginal applicators, similar differences in opinion related to applicator color were reported, with different colors being linked to personal preferences and associations and perceptions of the color relating to other personal experiences [42].

Preferences for the individual colors of the rings shown to participants also varied and additional colors were suggested. All example rings were pale in color, and women generally liked the low intensity of the ring colors, perceiving the lighter shades to be indicative of lower chemical content. However, some women suggested other possible ring colors, including options of more intense color varieties of what was presented to them (pink, yellow) in the FGDs.

Similarly, women felt their male partners may have different preferences for color. Some particular concerns women mentioned were that men might not accept products that were not white, as that is what they may be used to, and colored vaginal products could raise suspicion of possible bewitching. In contrast, other participants highlighted male partner preferences to colored male condoms. This may be related to the currently available South African public sector male condom, which is available for free. It was recently rebranded from “Choice” to “Max”, based on market research that confirmed that potential condom users wanted something new and more desirable [43]. Launched in 2015, the Max male condom is currently available in four color/scent combinations—red/strawberry scent in a red packet, yellow/banana scent in a yellow packet, purple/grape scent in a purple packet and plain latex color/unscented in a blue packet [44]. The South African public sector female condom has undergone similar rebranding and is currently available as “Maxima” in three color/scent combinations—red/strawberry scent in a red packet, yellow/vanilla scent in a yellow packet and plain unscented in a blue packet. A recent national survey has confirmed that these new brands are well liked and accepted [35].

Similar to color, preferences for scented vaginal products varied. Some women did not want to use products that contained scents, and these concerns were largely linked to perceptions that chemicals in the scents could cause vaginal irritation for themselves and their male partners [39]. Preference for intensity of scent was also related to perceptions as to whether more intense scents had higher chemical compounds and more likelihood of being unsafe. Other participants had positive attitudes towards scented products because they believed they would mask the “smell” of sex. Several participants used the example of the South African government colored and scented Max male condoms, and how they improved the smell of the environment during and after sex. This was also reported in a recent National Condom Survey in South Africa [44].

Preferences of the individual scents of the study rings differed across and between the three groups. Participants had strong opinions for which scents they preferred, and for scents that they disliked, often related to personal experiences and preferences.

Regarding the duration of scent, many participants felt that it should be retained over time, over a longer duration beyond the insertion. However, others raised concerns that the scent might be noticed by partners during sex, in particular if they had not told their male partner(s) that they were using the ring. Vaginal irritation was also mentioned as a concern if the scent remained over time. To further address the potential for scent retention within a vaginal ring device over time, it is necessary to understand the chemical composition of scents and the physicochemical processes underpinning molecular diffusion. Essential oils often comprise several hundred different chemical substances, mostly monoterpenes, sesquiterpenes and various oxygen-containing organic molecules (such as alcohols, phenols, aldehydes, ketones and esters). Generally, only a very small number of substances are present at concentrations above 5% within each oil, with most at significantly less than 1%. For example, the major chemical components of lavender essential oil are 1,5-dimethyl-1-vinyl-4-hexenylbutyrate (44%; 224.3 g/mol), 2,6-dimethyl-1,5,7-octatriene (25%; 136.2 g/mol), eucalyptol (7%; 154.3 g/mol) and camphor (4%; 152.2 g/mol) [45]. Chemical compounds must be sufficiently volatile to impart a smell or scent, since they must diffuse through the air to the olfactory system in the upper part of the nose. Therefore, essential oil components generally have low melting points (and commonly exist as liquids at room temperature) and lower relative molecular masses/volumes compared to drug substances (typically less than 250 g/mol, as illustrated in brackets above for lavender essential oil). By comparison, most steroid and antiretroviral drugs incorporated into vaginal rings for therapy are crystalline solids at room temperature and have relative molecular masses in excess of 250 g/mol (e.g., dapivirine 329.4 g/mol; ethinyl estradiol 296.4 g/mol; progesterone 314.5 g/mol). As such, it would prove challenging to maintain sustained or controlled release of scent components from a ring device; their high volatility and diffusivity would lead to rapid depletion from the device.

There was more agreement between women about preferred size/dimension of the vaginal ring. Participants generally preferred the “A” ring (Fig. 3) which—although having a slightly larger external diameter (59.6 mm) compared to B, C and D—had the smallest cross-sectional diameter (5.6 mm) of all the rings. However, several participants cautioned that their opinion may change if they were able to actually try the different sizes for fit and comfort. Differences in initial perceptions of size and actual fitting and use was reported by van der Straten, where women described the initial reaction to the dapivirine vaginal ring (size 56 mm external and 7.7 mm cross-sectional diameter) as intimidating [46]. However, after fitting the ring, almost all women were pleasantly surprised that they did not have a problem with insertion and did not feel the ring once fitted [22]. Flexibility of the ring was an important consideration in these discussions, with thicker rings perceived as less flexible and more difficult to insert. Participants who had used female condoms, made comparisons to the female condom internal ring that is used to insert the condom. Considerations of size and flexibility have been reported previously in vaginal acceptability studies [18, 34] with some women reporting they would prefer a smaller more flexible ring [22]. Although previous research has shown that women have been concerned that a ring could cause a blockage, e.g., during menses [47], this concern was not raised by participants in this study.

Participants also expressed opinions on partner preferences related to how size/dimension of the ring would impact on his sexual experience, and this has also been reported elsewhere [34]. There was some concern that the ring may directly impact on the size/tightness of the vagina, and if the partners penis would pass through the ring causing discomfort. Similar to participants own preference, size “A” or “D” were felt to be better for male partners.

Women participating in vaginal ring HIV prevention studies have frequently mentioned a preference for using a device without partner knowledge [26], although in a study with male partners they felt that men should be engaged in ring use decisions in order to facilitate trust and open communication in relationships [48]. Some participants in our study also discussed covert use in relation to all three ring attributes, with particular concerns about which sizes may be more noticeable during sex than others. Vaginal ring studies to date have only included white unscented products, and so the size of the ring has been the main concern related to ability to use covertly. In our groups, although scent was seen to be positive attribute by many women, some felt that if a scent continued during sex, this could be noticed by a partner and make it less likely to be covertly used. Similarly, women suggested a colored ring may raise more partner concern than a plain white ring, especially concerns of bewitching.

A wide range of preference was voiced for color and scent with some participants preferring products without any color or scent for themselves and their partners. Providing women and men choice in options for sexual and reproductive health products such as contraception has shown that increased choice increases overall uptake [49]. However, the range of options for a product such as the vaginal ring may be restricted by logistics and cost. Currently male condoms in the South African government public health sector include four options, including one male condom that has no color or scent, acknowledging that some people prefer unscented and uncolored products [44].

Other research notes the importance of understanding the socio-cultural context which may influence the uptake and use of products such as vaginal rings [50]. In this study, participants described that their male partners may be concerned about witchcraft if they were to see them using colored vaginal rings, similar to another study where male partners were initially concerned that the vaginal ring was a potion or magic snake [48]. Furthermore, participants in this study discussed the impact of vaginal ring use on virginity testing practices. Socio-cultural factors such as these influence women’s (and their male partner’s) preferences across and between communities. This highlights the importance of providing users with options and a range of products to cater for diversity within South Africa and sub-Saharan Africa more broadly, so that they can make choices to suit their individual circumstances.

### Limitations

Due to social distancing restrictions as a result of COVID-19, the groups comprised fewer participants than were originally anticipated. In spite of this, discussions were lively and richly detailed and participants were able to contribute and provide detailed accounts of preferences and acceptability. In addition, the perceptions were not based on actual use experience with the example products. Participants were only able to look at, feel and smell the example products. However, many participants had some experience with use and insertion of vaginal rings. The perceptions of product naïve participants were also important, since when products are being marketed there will be potential users who do not have product experience, and uptake will also be impacted by acceptability of the inexperienced users.

Ring color preference may be dependent on other factors not considered here, including age, culture, clinical indication. For example, would women’s color preferences be the same for vaginal ring devices for HIV prevention, estrogen replacement therapy and contraception?

We did not investigate preferences across different color intensities (saturations) and hues.

## Conclusions

In general, women’s preferences for ring color and scent were more diverse than for ring size. With respect to ring size, women were primarily concerned with rings being too large, both for personal fit and comfort and their partner feeling the ring during intercourse.

For some women, ring colors (aside from white) and scents were associated with chemicals, raising concerns of potential irritation for themselves or male partners. Conversely those that liked color and/or scent expressed strong personal preferences for the different colored and scented rings presented in the FGDs.

What is clear from the data is that women’s preferences around vaginal ring size, color and scent are personal and diverse. More research is needed to better understand these product attributes and, ultimately, offer women greater choice and options in the design of sexual and reproductive health products with a view to facilitating increased uptake, acceptability, and adherence.

## Data Availability

Data from the study will be made available on reasonable request from the authors. Further information/specifications for any of materials used to manufacture rings can be requested from Professor Karl Malcolm (k.malcolm@qub.ac.uk).
